# Rare Clinical Association between *Clostridioides difficile* Infection and Ischemic Colitis: Case Report and Literature Review

**DOI:** 10.3390/medicina57070705

**Published:** 2021-07-11

**Authors:** Elena Mirela Ionescu, Ana-Maria Curte, Andrei Ovidiu Olteanu, Carmen Monica Preda, Ioana Tieranu, Artsiom Klimko, Cristian George Tieranu

**Affiliations:** 1 Department of Gastroenterology, “Elias” Emergency University Hospital, 011461 Bucharest, Romania; dr.olteanuandrei@gmail.com (A.O.O.); cristian.tieranu@umfcd.ro (C.G.T.); 2 Department of Gastroenterology, “Carol Davila” University of Medicine and Pharmacy, 020021 Bucharest, Romania; carmenmonica.preda@gmail.com (C.M.P.); ioana.tieranu@drd.umfcd.ro (I.T.); klimkoartiom@gmail.com (A.K.); 3 Department of Pathology, “Elias” Emergency University Hospital, 011461 Bucharest, Romania; anma.pop@gmail.com; 4 Department of Gastroenterology, Fundeni Clinical Institute, 022328 Bucharest, Romania

**Keywords:** *Clostridioides difficile* infection, ischemic colitis, cause, effect

## Abstract

*Background and Objectives:* Gut microbiota plays an important role in the wellbeing of the host through different interactions between microflora constituents. In certain instances, *Clostridioides difficile* may pullulate, causing infection with associated colitis that may vary in terms of severity from mild disease to severe colitis, with increased associated mortality due to its complications. However, there are few literature data regarding the association between *Clostridioides difficile* and ischemic colitis. *Case report:* We report the case of a 30-year-old male patient, overweight, with impending dehydration, who presented with hematochezia and colicky abdominal pain, with positive fecal tests for the detection of *Clostridioides difficile* infection and endoscopic appearance suggesting ischemic colitis in the sigmoid and left colon, confirmed by computed tomography and histology. The patient was treated with oral Vancomycin, with resolution of symptoms, and was reevaluated through colonoscopy eight weeks after discharge, with endoscopic mucosal normalization and histological scarring process on biopsy samples. *Conclusion:* We report one of the few cases in the literature of ischemic colitis associated with *Clostridioides difficile* infection, with resolution of clinical, endoscopic, and histologic changes after specific treatment with oral Vancomycin suggesting a possible association between the two diseases. We also review the existing literature data regarding this comorbid association.

## 1. Introduction

There is growing evidence supporting the interaction between the host and gut microbiota which further promotes wellbeing and health. The main implications of microflora in the host–gut interaction resides in the process of short-chain fatty acids production through breaking down of indigestible dietary proteins and carbohydrates. Another major role of microbiota consists of vitamin production, ions absorption, and conversion of alimentary polyphenols into their active forms. Finally, microbiota plays a major role in the interaction with intestinal immune cells, generating signals and promoting cell maturation contributing to the normal immune system functionality [1,2,3].

*Clostridioides difficile* infection (CDI) has recently become a major healthcare-associated infection worldwide with great impact on healthcare systems as it evolves to a public health problem [4]. In fact, an analysis of death certificates in the United States of America identified CDI as the leading cause of diarrhea associated mortality, with an estimated death toll of 14,000 patients in 2007 [5]. Usually, antibiotic treatment is successful for primary CDI, but relapse is common, with recurrence rates ranging from 20% to 25% [4].

The pathogen—*Clostridioides difficile*, is present in the environment and has been identified on different surfaces such as pets, farm animals, food, soil, health care facilities, and even water. The disease may develop due to multiple factors, including but not limited to different drugs usage, especially antibiotics and proton-pump inhibitors (PPIs), which interfere with intestinal flora promoting colonization and altering the immune status in particularly prone patients with inadequate nutritional status [6].

Common risk factors associated with the development of CDI include prior hospitalization, advanced age, obesity, the use of medication such as antibiotics, proton-pump inhibitors and non-steroidal anti-inflammatory drugs [7,8,9,10]. Antibiotics that are commonly implicated in CDI include clindamycin, fluoroquinolones, cephalosporins and broad-spectrum penicillin [11].

On the other hand, ischemic colitis (IC) results from diminished blood flow to the bowel wall and is the most frequently encountered type of intestinal ischemia. The ischemic injury can result in variable degree of colonic wall damage, ranging from superficial injury to full-thickness necrosis and perforation [12]. IC mostly affects old female patients, and the clinical picture involves abdominal pain, diarrhea and hematochezia [13]. The clinical evolution is based on the classification proposed by Boley and Brandt, and is related to the level of bowel wall injury as follows: (1) reversible (submucosal bleeding); (2) transient colitis; (3) chronic segmental ischemic injury; (4) gangrenous colitis; (5) fulminant colitis [14].

Risk factors for the development of IC include advanced age, associated cardiac, metabolic and renal co-morbidities, vascular diseases (autoimmune, aneurysms, thrombotic states), smoking, and cocaine use, the latter especially in young adults [15,16,17]. There is a paucity of evidence reporting the simultaneous existence of both entities. We present a case where IC and CDI were diagnosed concurrently, in a patient with apparently no risk factor for any of the two diseases.

## 2. Case Report

A 30-year-old male patient without any history of diseases and without any prior treatments, was admitted in the Gastroenterology Department of the “Elias” Emergency University Hospital in Bucharest for hematochezia—three stools in the last 24 h, and colicky abdominal pain in the left lower quadrant. He denied experiencing unexplained weight loss or having similar symptoms before the present episode. His family history was negative for inflammatory bowel diseases and colorectal cancer. He denied the use of medication, drugs, alcohol consumption or smoking prior to hospital admission but his lifestyle anamnesis revealed an insalubrious workplace in a construction site with severely altered dietary habits regarding regular meals and adequate hydration. He was overweight, with a Body mass index (BMI) of 29 kg/m^2^.

Clinical examination upon admission revealed normal hemodynamic and respiratory parameters, normal temperature, with pain located in the lower left quadrant upon palpation but without any peritoneal signs.

Laboratory data showed leukocytosis with neutrophilia (16,000/μL, reference range 4600–10,200/μL), elevated C reactive protein at 4-times the upper limit of normal (22 mg/dL, reference range < 5 mg/dL), and low serum iron but without anemia. The stools were negative for parasites. Although the stool culture was negative, enzyme immunoassays for toxins A, B, and glutamate dehydrogenase (GDH) for the detection of CDI were all positive. Serum sodium level was 146 mmol/L (reference range 136–145 mmol/L) while serum osmolality 299 mOsm/kg. (280–300 mOsm/kg). The patient was started on oral Vancomycin 125 mg every 6 h associated with rehydration with intravenously administered crystalloids.

**Figure 1:**
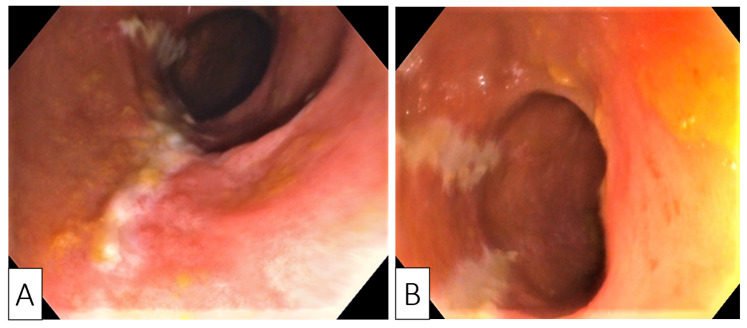
Endoscopic images showing longitudinal (**A**) and parallel (**B**) superficial ulcerations with surrounding mucosal edema and disappearance of colonic wall visible vascularization.

Despite no family history, sigmoidoscopy was performed to screen for inflammatory bowel disease or colorectal cancer, as there were no major risk factors for the development of CDI (e.g., use of antibiotics or prior hospitalization). Endoscopy revealed erythematous mucosa with longitudinal and parallel superficial ulcerations, in addition to luminal narrowing spanning from the junction of the sigmoid and descending colon. This narrowing precluded the advancement of the endoscope further—the appearance was highly suggestive for ischemic colitis (Figure 1A,B). 

The procedure was prematurely abandoned due to the risk of perforation in this setting and biopsies were taken from the areas of ulceration. 

**Figure 2:**
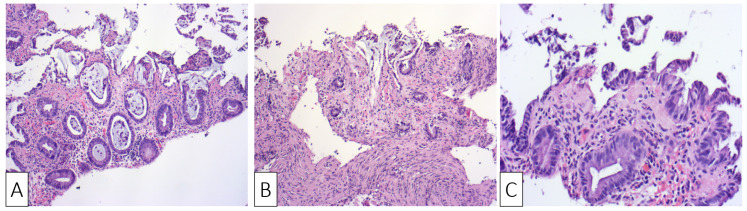
H&E ×10 Sigmoid mucosa at initial admission of the patient showing: (**A**) sloughing, epithelial regenerative changes, and focal hemorrhage with mild inflammatory changes; (**B**) sloughing, epithelial regenerative changes, and stromal hyalinization; (**C**) surface erosions and lamina propria hyalinization.

The histological examination confirmed the diagnosis of ischemic colitis describing colonic mucosa with surface epithelial degeneration, areas of mucosal necrosis and loss of superficial portions of glands (“withering crypts”). Lamina propria showed hyalinization, capillary congestion, focal hemorrhage, and reduced acute inflammation. The crypts were mucin depleted and the lining epithelium presented nuclear reactive changes consisting of nuclear hyperchromasia and enlargement, along with an increased number of mitotic figures (Figure 2A–C).

**Figure 3:**
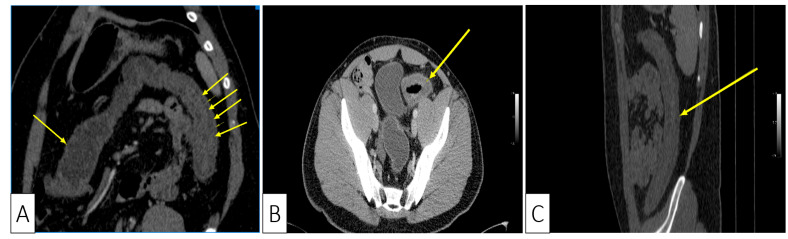
Abdominal CT showing: (**A**) Coronal plane, oblique reconstruction image showing the difference between the normal wall (single arrow at the transverse colon) and the thickened pathological wall of the left colon (multiple arrows); (**B**) Axial plane, venous phase, showing stratified wall thickening of the proximal sigmoid; (**C**) Sagittal plane, showing the left colonic involvement with symmetrical wall thickening.

Consequently, we continued to investigate the patient with a computed tomography (CT) which showed a symmetrically thickened colonic wall corresponding to the descending and sigmoid segments, without obvious stenosis and without dilation of the proximal segments. The colonic vascularization on CT scan was negative for arterial or venous thromboses and the presence of the Comb sign was supportive of a local inflammatory process (Figure 3A–C).

No supplementary treatments were added because the patient’s evolution was rapidly trending towards normalization of stools consistency, with disappearance of abdominal pain and rectal bleeding on the second day of treatment. The patient was discharged after 14 days of treatment with recommendations to perform a review colonoscopy in eight weeks. Additionally, he was advised to perform thrombophilia screening in an outpatient clinic. 

He received no further medications after discharge. 

**Figure 4:**
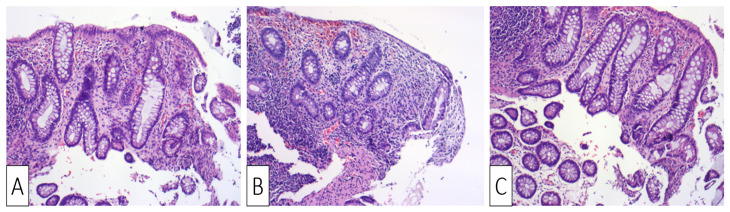
H&E ×10 Sigmoid mucosa at follow-up following treatment: (**A**) Sigmoid mucosa showing mild distortion of crypts and lamina propria densification.; (**B**) sigmoid mucosa showing distortion of crypts and lamina propria densification; (**C**) sigmoid mucosa showing lamina propria densification (top right) compared to normal stroma (bottom left).

Upon readmission, the patient was clinically normal, his laboratory tests were normal, his weight continued to be stable, and his thrombophilia screening tests were negative. The colonoscopy showed a normal mucosa, with resolution of the prior stenosis, without any lesions throughout the entire colon and the last 15 cm of the terminal ileum. Biopsies were obtained from apparently normal mucosa at the site of prior ulceration, showing areas of lamina propria densification and residual predominantly lymphoplasmacytic inflammation, along with focal architectural changes (Figure 4A–C).

## 3. Discussions

The evolution process of societies caused by the industrial revolution has contributed to enormous advance in medicine with concurrent increase in life expectancy, but it has also altered disease evolution patterns in these populations. The modern lifestyle leads to the eradication of nonpathogenic bacteria through limited beneficial bacteria intake, along with ageing process and dietary changes as contributing factors for the development of altered gut microbiota [18].

The individual flora diversity is influenced by multiple circumstances such as acute diarrhea, PPIs, NSAIDs and antibiotic treatments, or to a lesser extent, dietary excess. These factors can alter gut microbiota, either alone or in combination, generating changes in the intestinal microenvironment that make the host susceptible to the action of pathogenic agents. 

The most studied aforementioned factor is represented using antibiotics which have been shown to influence gut microbiota through either interference with mutualistic interaction between different community members, or direct toxic effect on others [19]. Thus, the loss of the intestinal environmental microbial equilibrium created the premises for the development of bacterial species such as *Clostridium difficile.*

Until now, two cases have been described in the literature with ischemic colitis and CDI colitis in the same patient.

Most frequently, ischemic colitis is caused by non-occlusive ischemic injury to the bowel wall through sudden decrease in blood supply in the small vessels of the colon, usually secondary to a low circulating volume state [20,21]. There are several well-established risk factors that predispose towards the development of ischemic colitis, such as atherosclerosis, aortic surgery, oral contraceptives, hereditary coagulopathies, cocaine abuse, infectious colitis caused by Cytomegalovirus (CMV) and *Escherichia coli* [22]. In our patient, none of the above-mentioned predisposing factors were encountered. 

Regarding the anatomical distribution of ischemic colitis, the disease has been classified as left- and right-sided. The former is usually associated with diminished blood flow, coagulopathies, cardiac and aorta surgery, while the latter is generally caused by superior mesenteric artery obstruction [23]. Our patient presented with a left-sided ischemic colitis in the absence of any history of surgery and testing negative for thrombophilia, with the only remaining causative factor related to dehydration.

The set of predisposing factors for onset of ischemic colitis early in life has been studied and found to be distinct from disease affecting older patients—smoking was found to be the most important risk factor. 

Dehydration is defined by an increase in serum osmolality above normal values (275–295 mOSm/kg) and is classified into impending and current based on the severity of serum osmolality increase (impending dehydration 295–300 mOsm/kg, current dehydration > 300 mOsm/kg) [24,25]. It results mainly through insufficient fluid intake and excessive loss in vomiting and diarrhea. In our case, the impeding dehydration at admission might have been caused by insufficient intake developing intro current dehydration through diarrhea. 

There has been identified an entity mimicking ischemic colitis in long-distance runners—called “runner’s colitis”, which is also presumed to be an ischemic colitis caused by dehydration [15,26]. In our case, the patient denied smoking and did not declare any recent strenuous physical effort which may have contributed to dehydration. 

In stark contrast, CDI has been a topic of rigorous research and risk factors, such as recent hospitalization and prior antibiotic use, are well-described risk factors for the development of colitis. However, none of these factors were present in our patient. 

The deleterious progression to severe colitis in CDI is well-described in the literature and involves a mixed infectious and ischemic insult to the colonic mucosa. The mechanism implicates a stepwise exacerbation secondary to dehydration and hypotension precipitated by severe diarrhea induced by CDI, which leads to either global or localized bowel ischemia. This contributes to a more pronounced systemic inflammatory response and subsequently, fulminant colitis [27,28,29].

To further explore the co-incidence of ischemic and pseudomembranous colitides, we conducted a literature review using the PubMed and Scopus databases. A set of relevant keywords were identified either within the title and/or abstract or as medical subject heading (MeSH) terms. Per our selection strategy, we identified seven published case reports which presented patients with closely associated pseudomembranous and ischemic colitis or pseudomembranous colitis with histologically confirmed ischemic changes. However, despite the presence of pseudomembranes and high index of clinical suspicion for CDI, we excluded 5 cases due to lack of definitive confirmation for CDI. A mandatory inclusion criterion for our review was the presence of confirmed CDI via identification of stool pathogens on stool studies or polymerase chain reaction. A total of two case reports and three patients were included in the review and our findings are presented in Table 1.

The clinical picture in all three cases was expectedly dominated by colicky abdominal pain and bloody diarrhea. In one case, the authors mentioned the D-1 toxin assay was strongly positive at admission but became negative two days after hospitalization. The presumed etiology was Cefteram use for several days to treat gingivitis. Lymphocyte stimulation test with Cefteram was negative prior to onset of symptoms, making a hypersensitivity reaction or antibiotic-associated hemorrhagic colitis an unlikely diagnosis. The authors suggest that disease severity may be related to difference in host response, as well as qualitative and quantitative variations in C. difficile toxins. In the second case, the patient was being treated with anti-rejection therapy for severe acute rejection that arose two weeks after the transplant. The patient experienced seroconversion of CMV with a subsequent tissue-invasive vasculitis of the colon; however, there was no response to ganciclovir. Further workup yielded positive C. difficile toxins and uncovered severe ischemic colitis which was treated with subtotal colectomy and Hartmann’s procedure. Unfortunately, the patient’s status continued to deteriorate, and she passed away two weeks later. Despite CDI being the most common complication of renal transplant, this case demonstrates that in selected patients, CDI with superimposed ischemic colitis can lead to a fatal outcome.

It is important to underline that in both previous cases, there was a clear precipitating factor, such as previous antibiotic use or hospitalization. We can only postulate the mechanism underlying the findings in our case, as local inflammation secondary to CDI might have determined local thrombosis and secondary ischemic changes. Preliminary research suggests ischemic colitis may be a risk factor for CDI; however, it is unclear if the opposite applies [32].

Still, we did not identify any major cause for the development of CDI-associated colitis in our patient, and in this setting, we can always question what the cause was and what was the effect. The inflammation secondary to CDI colitis in the setting of impending dehydration could have precipitated local ischemia. Another theory resides on an initial ischemic colonic injury causing diarrhea associated with CDI through a mechanism based on dysbiosis, similar to the mechanism seen in patients suffering from ulcerative colitis [33].

Nevertheless, the rapid clinical improvement after appropriate treatment, a lack of other identifiable causes for ischemic colitis, and the normalization of colonic mucosa on follow-up colonoscopy in the absence of other treatments, favor the enteric pathogen as main cause for the ischemic episode.

## 4. Conclusions

We present one of the few literature reports linking ischemic colitis to CDI, in a clinical and evolutive picture favoring a cause-effect relationship towards the infection as the triggering factor for colonic ischemia. Ischemic colitis might be a complication of CDI and should be suspected especially in complicated cases of CDI. This observation needs validation which might come from larger cohorts of CDI suffering patients.

## Figures and Tables

**Table 1:** Case reports of patients with co-incidence of confirmed CDI and IC.

Author and Year	Gender and Age (Years)	Symptoms	Stool Study Results	Presumed Etiology	Endoscopy Findings	Histology Findings	Treatment and Outcome
Our patient	Male, 30	Colicky left lower quadrant abdominal pain and bloody diarrhea	Negative fecal culture, positive toxins A, B, and GDH	Idiopathic	Several superficial longitudinal ulcerations with luminal narrowing spanning from sigmoid to the descending colon	Desquamation of superficial epithelium with focal necrosis, loss of superficial glands, edematous and necrotic lamina propria	Oral Vancomycin, symptoms resolved within two days of hospitalization
Okada et al., 1997 [30]	Male, 52	Crampy lower abdominal pain followed by bloody diarrhea	Positive fecal culture and strongly positive D-1 toxin	Three-day use of Cefteram for gingivitis	Edematous mucosa with multiple longitudinal shallow ulcers, erosions, and luminal narrowing	Desquamation of superficial epithelium with crypt degeneration, fibrinous exudates in lamina propria, and mild neutrophilic infiltration	Supportive treatment, symptoms resolved by third day of hospitalization
Veroux et al., 2007 [31]	Female, 42	High fever, diffuse abdominal pain with tenderness and bloody diarrhea	Positive toxins	Two weeks after renal transplant; CDI colitis was complicated by CMV colitis	Edematous mucosa with patchy erythema and longitudinal ulceration	Mucosal inflammation, hemorrhage, with leukocytoclastic vasculitis and lymphoplasmacytic perivascular infiltrate	Metronidazole and oral Vancomycin for seven days with no improvement; patient underwent subtotal colectomy but passed away two weeks later
